# *In situ* dynamic observations of perovskite crystallisation and microstructure evolution intermediated from [PbI_6_]^4−^ cage nanoparticles

**DOI:** 10.1038/ncomms15688

**Published:** 2017-06-21

**Authors:** Qin Hu, Lichen Zhao, Jiang Wu, Ke Gao, Deying Luo, Yufeng Jiang, Ziyi Zhang, Chenhui Zhu, Eric Schaible, Alexander Hexemer, Cheng Wang, Yi Liu, Wei Zhang, Michael Grätzel, Feng Liu, Thomas P. Russell, Rui Zhu, Qihuang Gong

**Affiliations:** 1 State Key Laboratory for Artificial Microstructure and Mesoscopic Physics, Department of Physics, Peking University, Beijing 100871, China; 2 Collaborative Innovation Center of Quantum Matter, Beijing 100871, China; 3 Materials Sciences Division, Lawrence Berkeley National Laboratory, Berkeley, California 94720, USA; 4 Laboratory of Photonics and Interfaces, Institute of Chemical Sciences and Engineering, École Polytechnique Fédérale de Lausanne, Lausanne CH-1015, Switzerland; 5 Advanced Light Sources, Lawrence Berkeley National Laboratory, Berkeley, California 94720, USA; 6 Molecular Foundry, Lawrence Berkeley National Laboratory, Berkeley, California 94720, USA; 7 Advanced Technology Institute, University of Surrey, Guildford, Surrey GU2 7XH, UK; 8 Department of Physics and Astronomy, and Collaborative Innovation Center of IFSA, Shanghai Jiaotong University, Shanghai 200240, China; 9 Department of Polymer Science and Engineering, University of Massachusetts, Amherst, Massachusetts 01003, USA; 10 Collaborative Innovation Center of Extreme Optics, Shanxi University, Taiyuan, Shanxi 030006, China

## Abstract

Hybrid lead halide perovskites have emerged as high-performance photovoltaic materials with their extraordinary optoelectronic properties. In particular, the remarkable device efficiency is strongly influenced by the perovskite crystallinity and the film morphology. Here, we investigate the perovskites crystallisation kinetics and growth mechanism in real time from liquid precursor continually to the final uniform film. We utilize some advanced *in situ* characterisation techniques including synchrotron-based grazing incident X-ray diffraction to observe crystal structure and chemical transition of perovskites. The nano-assemble model from perovskite intermediated [PbI_6_]^4−^ cage nanoparticles to bulk polycrystals is proposed to understand perovskites formation at a molecular- or nano-level. A crystallisation-depletion mechanism is developed to elucidate the periodic crystallisation and the kinetically trapped morphology at a mesoscopic level. Based on these *in situ* dynamics studies, the whole process of the perovskites formation and transformation from the molecular to the microstructure over relevant temperature and time scales is successfully demonstrated.

## Abstract (web summary)

The photovoltaic performances of perovskite materials are strongly influenced by their crystallinity and film morphology. Here, the authors investigate the formation and morphology evolution mechanisms of lead halide perovskites and reveal that bulk polycrystals grow from intermediate [PbI6]^4^^−^ cage nanoparticles.

Thin-film photovoltaics that use hybrid lead halide perovskites as photon-conversion layers have been a rapidly developing technology, offering remarkable device performance at low production costs (https://www.nrel.gov/pv/assets/images/efficiency-chart.png)^1^,^2^,^3^,^4^. Tremendous efforts have been devoted into improving the device power conversion efficiency^5^ and long-term stability^6^, and notable progresses have been achieved. 2However, the device performance is strongly influenced by the perovskite crystallinity and film morphology. Many of the optoelectronic properties of perovskites such as light harvesting, charge carrier transport and diffusion can be markedly influenced by the crystallisation and morphology of perovskites^7^,^8^. Although the correlations between material quality and device performance have been investigated widely, the in-depth understanding of the crystallisation kinetics and growth mechanism of the perovskites, especially the sight into material transformation from precursor solution to solid phase in a continuous manner has not been revealed.

The hybrid lead halide perovskite precursor is a low-viscosity solution that can form colloidal aggregates in solution and take complicated nucleation and growth pathways during drying process^9^. The solvent removal rate, diffusion of the constituents and crystallisation processes are critical in generating the kinetically trapped morphologies of the perovskite films. Various factors, such as lead sources^10^, precursor solvents^11^ and assisted additives^12^ affect not only the crystallisation but also the microstructure formation in perovskite thin film formation. Many studies on the *ex situ* observation^13^ of the crystallisations and the effects on the surface morphology have been reported. For example, the cuboid size of perovskite CH_3_NH_3_PbI_3_ could be controlled by the dipping time of the PbI_2_ substrate into CH_3_NH_3_I (MAI) solution and the MAI concentration^14^. A series of scanning electron microcopy (SEM) images of the statistic perovskite samples could correlate the surface morphology changes and the cuboids crystallisation behaviour under various growth conditions. It is well known that an incontestable limitation of these *ex situ* studies are the insufficient information collection to further scrutinize perovskites growth mechanism. In addition, there are also some literatures reporting the chemical pathways from the plumbate intermediate^15^,^16^ or metastable phase^17^,^18^ to the certain perovskite crystal identified by the *in situ* X-ray diffraction. However, the time evolution of crystallisation and phase transition in such *in situ* cases usually begins with the prepared solvent-intermediated complex film, not the pristine mixed solution in our work. For these kinds of methodologies without the analysis of the pure precursor, they cannot draw a conclusion on whether the perovskite crystal growth begins in the solution phase or in the solid phase. Even though there are a few reports observing the perovskite crystallisation from drop-casting solution to dry film^19^, however, the full transformation process to pure perovskite was not achieved, and the clear formation mechanism was still indistinct with existence of by-products in the final dried film. Hence, a comprehensive investigation of perovskites crystallisation dynamics and morphology evolution from the very original precursor to the solid-phase crystals *in situ* and in real time is critically important to further improve the material quality and the corresponding device performance.

Here, we present a study, apply some *in situ* on-line characterisation techniques to systematically investigate the perovskites crystallisation kinetics and growth mechanism from the precursor solution to the uniform film. The use of *in situ* grazing incidence X-ray diffraction (GIXD) with *in situ* Fourier transform infrared spectroscopy (FTIR) reveals the crystalline formation and chemical composition reactions of perovskite materials over relevant time and temperature scales. A nano-assemble model from perovskite intermediate nanoparticles to bulk polycrystals is proposed to understand perovskite formation at a molecular- or nano-scale. The temperature-dependent crystallisation process also makes effects on the final film morphology. The time evolution of the film morphology under *in situ* heating is obtained by the compound optical microscope. The formation of periodic patterns during the growth process is observed which could be attributed to a competition between the linear growth of the crystals and a depletion of crystallisable material. The crystallisation-depletion mechanism is developed to elucidate the periodic nucleation and the kinetically trapped morphology at a mesoscopic level. Based on these dynamics studies, the whole process of the perovskites formation from molecular to microstructure is successfully demonstrated.

## Results

### Perovskites crystallisation and structure transformation

**Figure 1:**
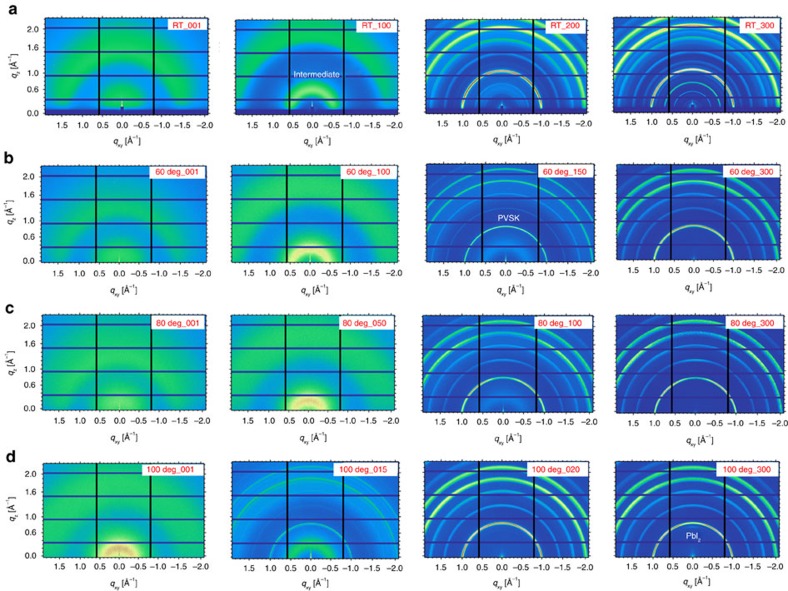
GIXD profiles of printing perovskites. 2D grazing incidence X-ray diffraction (GIXD) images at different stages of drying along with time annealing at different temperatures (300 frames): (**a**) room temperature (RT), (**b**) 60 °C, (**c**) 80 °C and (**d**) 100 °C (°C is short as deg).

All GIXD measurements were performed on beamline 7.3.3 at Advanced Light Sources of Lawrence Berkeley National Laboratory with critical methods to obtain the perovskites structure formation and crystal growth in real time. To *in situ* monitor the whole evolution process at the very beginning with the precursor solution, a compatible mini-slot-die printer^20^ was integrated with on-line GIXD, as shown in Supplementary Fig. 1. For the traditional spin-coating technique^21^, most of the deposition solution is ejected during rotation and the subsequent thermal annealing process begins with the intermediated complex film, hindering the capture of the whole crystallisation process. The *in situ* printing experiment was conducted in the helium atmosphere to reduce the air scattering. The perovskite precursor solution was prepared by mixing 0.4 M lead acetate (Pb(Ac)_2_) and 1.2 M methylammonium iodide (MAI) in anhydrous *N*,*N*-dimethylformamide (DMF). A PEDOT:PSS-coated silicon wafer was used as the sample substrate. Different substrate temperatures were adjusted to control the kinetics of crystallisation. A series of 2D GIXD images at different stages of drying are shown in Fig. 1 and Supplementary Fig. 2. In a starting solution, the mixed precursors are essentially point scatters dissolved in DMF, where the liquid scattering from DMF dominates, with a diffuse reflection at *q*∼1.5 Å^−1^ being evident. As the solvent, an intermediate forms formed characterized by a peak with a spacing of ∼1 nm (*q*∼0.6 Å^−1^), which then transformed into the perovskite crystallites. The kinetics of this structural transformation can be controlled by the substrate temperature and solution concentration.

**Figure 2:**
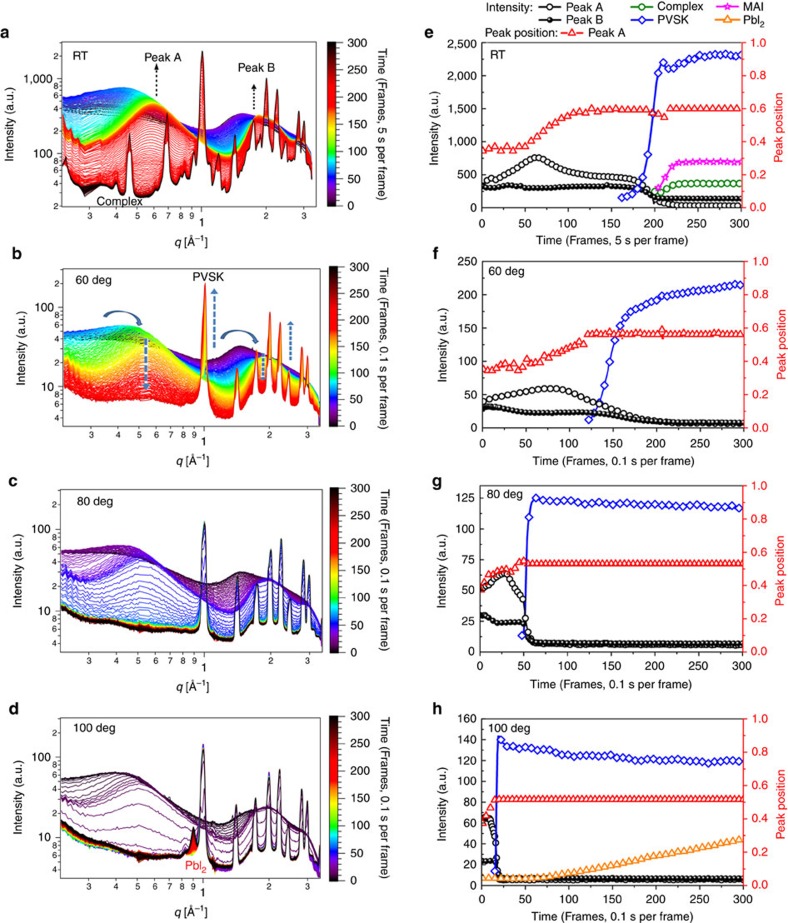
*In situ* integral GIXD profiles of printing perovskites. (**a**–**d**) *In situ* integral GIXD profiles at various temperatures along with time (300 frames) at (**a**) RT, (**b**) 60 °C, (**c**) 80 °C and (**d**) 100 °C. (**e**–**h**) The diffraction peak intensity and the peak position of the characteristic peaks at different temperatures: (**e**) RT, (**f**) 60 °C, (**g**) 80 °C and (**h**) 100 °C. The intensity of MAI and final complex adduct at RT was doubled to show clear evolution process. Peak A is noted as the broad peak shifting from 0.4 Å^−1^ to 0.6 Å^−1^, and Peak B is short of the broad peak shifting from 1.5 Å^−1^ to 2.0 Å^−1^. The peak shift process is shown in Supplementary Fig. 3. MAI is short of CH_3_NH_3_I, Complex is abbreviated of the MAI·PbI_2_·DMF adduct at RT. The abbreviation of CH_3_NH_3_PbI_3_ is PVSK.

The time evolution of perovskite crystallites formation was captured by time-resolved GIXD measurements at room temperature (RT), 60, 80 and 100 °C shown in Fig. 2a–d (2D intensity-time colour mappings are shown in Supplementary Fig. 3). The peak intensity, peak position and the peak area shown in Fig. 2e–h were calculated from the characterized peaks of GIXD through Multi-peak Mode fitting. A quite slow drying process was observed in the RT condition. At RT, a broad diffuse reflection at *q*∼0.4 Å^−1^, characteristic of the scattering arising from the dissolved components, was observed initially (5 s per frame). With time, the reflection gradually shifted to higher q, indicating the formation of aggregates (intermediate state of order) in the solvent that were the precursors to form the crystals. As the solvent evaporated, the reflection decreased in intensity, with the appearance of the cubic (100) plane reflection at 1.0 Å^−1^ of perovskite (CH_3_NH_3_PbI_3_, abbreviated as PVSK) observed. The crystalline reflections from the perovskite intensified and sharpened as crystallisation proceeded. In the high q region, a broad diffraction peak (1.5–2.5 Å^−1^) was seen initially, arising from the average intermolecular distances between solvent molecules. This broad peak may also be the form factor of the intermediates formed prior to perovskite crystallisation. As the DMF continued to evaporate, high ordered reflection characteristics of the perovskite crystals lattice plane dominated. The reflection characteristics of the perovskite crystals increased as the volume fraction of the crystals increased, whereas the reflection at 0.6 Å^−1^ decreased, implying that the precursor aggregates were transformed during the crystallisation, as shown in Figs 1 and 2a–d. As can be seen, both the 0.6 Å^−1^ intermediate peak (abbreviated as Peak A) and 1.8 Å^−1^ peak (abbreviated as Peak B) in Fig. 2 at RT show a reduction roughly at frame 180 (∼900 s), accompanied with the rapid perovskite crystal growth. Perovskite precursors in solution^22^ are charged species, including an octahedral [PbI_6_]^4−^ centre and other cooperative ions. Thus, the 0.6 Å^−1^ diffraction peak was assigned to the Pb^2+^ and its surrounding ions and molecules and the formation of an ion cage by electrostatic forces. The outmost shell of the ion cage is surrounded by DMF molecules. During solvent evaporation, the inter-cage distance gradually decreased, as evidenced by the scattering at low q (peak shifting from 0.4 Å^−1^ to 0.6 Å^−1^), and then close packs to give a 0.6 Å^−1^ steady peak. After the material transformation at RT, two peaks located at 0.48 Å^−1^ and 0.70 Å^−1^ appeared, arising from the MAI·PbI_2_·DMF adduct (abbreviated as Complex in Fig. 2a–d) and MAI crystallites (Supplementary Fig. 4) with an intensity ∼10% that of the major perovskite peak. The precursor–solvent adduct was also described in the previous reports^23^.

When a higher thermal annealing temperature was used, the film drying was much more rapid, and thus, a shorter X-ray exposure time (0.1 s per frame) was used. As seen from the 60, 80 and 100 °C experiments in Fig. 2e–h (blue curves), the structural transformation occurred more rapidly, 12.5, 5 and 1.8 s, respectively. It should be noted the MAI·PbI_2_·DMF adduct complex was not observed in the final product at elevated temperatures, indicating instability at these higher temperatures. At 100 °C, a prolonged heating for perovskite film led to PVSK decomposition and PbI_2_ formation, as evidenced by the appearance of the PbI_2_ (001) peak at 0.9 Å^−1^ peak (∼5.34% in amount comparing to perovskite roughly estimated by the characteristic peak area ratio). Further increasing heating temperature to 120 °C led to the formation of more PbI_2_, and this process was time-dependent, as seen from Supplementary Fig. 2 and Supplementary Fig. 5. Thus, reducing heating time and increasing the drying speed can be a viable route to kinetically trap the structure in a stable perovskite form. We also developed printing in parallel with a hot air quenching process (HAQ, 180 °C hot air) to continue increase the crystal growth speed, in which the heating and drying can be completed within several seconds and PbI_2_ formation can be effectively suppressed. However, this process was too fast to be probed in the current GIXD system.

The crystal sizes determined from a Debye-Scherer analysis of the reflection are plotted in Supplementary Fig. 6 and Supplementary Note 1, from which detailed crystal growth kinetics can be studied. The rate of crystallisation was modelled using the Johnson-Mehl-Avrami model ((*χ*(*t*)=1−exp (−*β*^*n*^)^24^,^25^, where *χ*(*t*) is the transformed fraction of the material, *t* is time, *β* is a state property which is independent of the time/temperature path, and *n* is the growth exponent) combined with the nucleation and growth models. Using the perovskite (100) peak area to determine χ(*t*) and the kinetic formula^26^: ln (*t*_χ2_−*t*_χ1_)=*E*_a_/RT−ln κ_0_+ln (*β*_χ2_−*β*_χ1_), where *E*_a_ is the effective activation energy, R is the gas constant, T is the temperature, and κ_0_ is a rate constant pre-factor. The calculated value of *E*_a_ was 81.4 kJ mol^−1^, which is lower than 86.6 kJ mol^−1^ of the lead chloride precursor and 97.3∼110 kJ mol^−1^ of lead iodide precursor^18^,^27^, indicating the faster crystallisation and film formation for lead acetate precursor. Also, the value of *E*_a_ for lead acetate precursor from slot die printing in our research is larger than 67.5 kJ mol^−1^ obtained from traditional spin coating^27^. This could be mainly because of the different thermal kinetics of different procedure as well as the different sample ambient environments such as humility and so on. The plots along with the fittings are shown in Supplementary Fig. 7. The transformation time (ln (*t*_χ2_−*t*_χ1_)) is inversely proportional to T, thus the perovskite transformation time decreases dramatically with the increasing temperature. This is also consistent with our experiment results.

Perovskite chemical structure evolution at RT was studied using *in situ* FTIR. Supplementary Fig. 8 shows the FTIR spectra of pure DMF, MAI, HAc (acetic acid, CH_3_COOH) and the perovskite films. The N-H stretching vibration of MAI (∼3030–3300, cm^−1^), the O-H stretching vibration of HAc (freedom 3500∼3550, cm^−1^, H-bonded 2500∼3300, cm^−1^), the N-H stretching vibration of DMF (3300∼3450, cm^−1^), the C-H symmetrical and asymmetrical bending vibrations (1580∼1750, cm^−1^) and the C=O stretching vibration of DMF (1650∼1700, cm^−1^ ) were used to track the changes in the chemical constitutions of the perovskite film^28^,^29^,^30^. From 1 to 13 minutes, the O-H band in HAc and N-H band in DMF deceased. The stretching vibration of COO-H disappeared after 7 min and the N-H band of DMF disappeared after 13 min. Thus HAc evaporated more rapidly than DMF and no HAc residue was left in fully dried film. The final spectrum after 16 min was a combination of bands from the perovskite and MAI at the stretching vibration of the N-H band. In addition, the dried film still had strong symmetric and asymmetric C-H bending vibrations from DMF. The C=O stretching vibration of DMF also shifted from 1670, cm^−1^ to 1650, cm^−1^ along with a decrease of the peak intensity. These chemical signatures indicated that DMF could be incorporated into lead-based crystalline species^31^, leading to the formation of a MAI·PbI_2_·DMF adduct complex, which gave rise to the low q diffraction peak in GIXD. Thus, the perovskite film formation at different temperatures can be described in the following chemical equations:


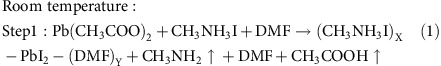



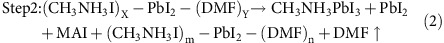



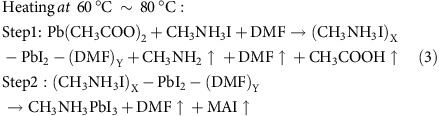



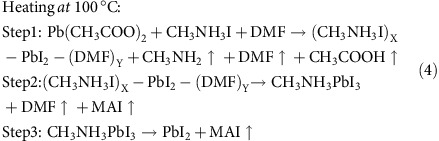


**Figure 3:**
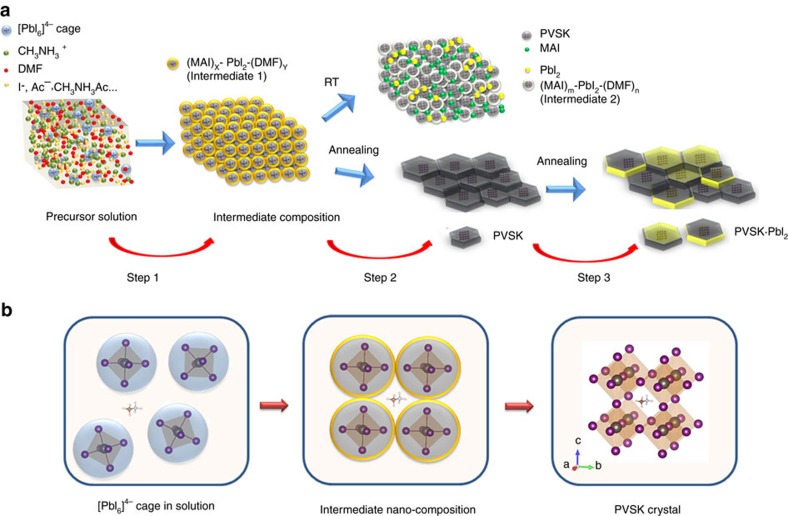
The nano-assemble model of perovskite crystallisation. (**a**) The nano-assemble model of the perovskite from the precursor solution to the final polycrystalline film, and the decomposition under high-temperature annealing. (**b**) The detailed crystal growth process from the [PbI_6_]^4−^ cage to intermediate nano-composition, and finally to the perovskite crystal.

The combined GIXD and FTIR characterisations revealed the detailed structure transformation of perovskite crystals. Figure 3a presented the nano-assemble model of the perovskites from the pristine precursor solution to perovskite polycrystals. Figure 3b shows the detailed perovskite crystal growth via the [PbI_6_]^4−^-centred nanoparticles. In precursor solution, the Pb(Ac)_2_ and MAI were dissolved in DMF. Solvent evaporation led to the formation of Pb-centred ion-cages (CH_3_NH_3_I)_X_-PbI_2_-(DMF)_Y_ (abbreviated as intermediate in Fig. 1a, marked as Peak A in Fig. 2e–h) as shown by equation (1). This ion cage is stable at room temperature and when the cage concentration increases to a point where the cages are forced into contact, they start to transform into more stable perovskite crystals with PbI_2_, MAI and another intermediate (CH_3_NH_3_I)_m_-PbI_2_-(DMF)_n_ (abbreviated as Complex in Fig. 2e–h). The ion cage should be centred with a [PbI_6_]^4−^ octahedron covered with MAI and DMF shells, with a diameter of ∼1 nm. The Lewis acid PbI_2_ provided a driving force to interact with the electron pair in DMF (oxygen atom) and MAI (iodide ion) to stabilize the product. At elevated temperature, the rapid decomposition of (CH_3_NH_3_I)_X_-PbI_2_-(DMF)_Y_ led to a higher yield of perovskite crystals with much less PbI_2_ and MAI by-products, and thus, high-temperature processing is beneficial in thin film fabrication. At even higher temperatures, the perovskite will be not stable and decompose into PbI_2_ and MAI (100 °C and above) with prolonged heating.

### Microstructure growth and film morphology evolution

**Figure 4:**
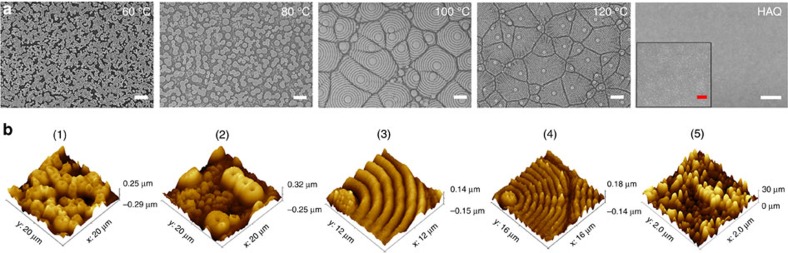
SEM and AFM images of perovskite films. The scanning electron microscope (SEM) (**a**) and topographic atomic force microscope (AFM) images (**b**) of perovskite films based on various heating temperatures, the printing speed was 30 mm s^−1^ and the distance between the slot die head and the substrate was 0.2 mm. The white scale bars in **a**(1–4) are 10 μm. The white scale bar in **a** (5) is 2 μm, whereas the red one in the corresponding magnifying image is 500 nm.

The perovskite film morphology has a vital role in optoelectronic devices. The film drying kinetics as well as the crystallisation dynamics directly from the liquid precursor is quite different from processes like solvent exchange^32^ and vapour deposition^33^. The temperature control^34^ of thin film drying is the most accessible way to control the morphology in a continuous fabrication process. Figure 4 and Supplementary Figs 9-11 show the morphologies of printed perovskite thin films at different temperatures and drying speeds. As seen from the SEM and atomic force microscopy (AFM) images, low-temperature processed perovskite thin films were dominated by large irregular crystalline grains with domains tens of microns in size (Supplementary Fig. 9). The non-continuous passivation of perovskites layers is not favourable for device fabrication. The 60 °C-processed thin film with smaller size droplets were still not continuous. At 80 °C, the film showed slightly enlarged droplets, many of which merged together to form larger domains. When the heating temperature was increased to 100 °C, a highly regular, banded topography with a period of ∼2 μm was seen (Supplementary Figs 10 and 11). The height variation of this periodic structure at the surface, that is, the amplitude, was ∼200 nm (Supplementary Fig. 10). At the centre of these banded structures, a plateau consisting of nanoscopic crystallites was also observed. These structures are very much akin to spherlitic textures seen in semi-crystalline polymers and metals^35^,^36^,^37^, where a radial growth of crystals emanates from a nucleus, and as the crystals grow outward, the growth fronts impinge on each other, arresting the crystallisation, forming the polygonal textures. The sizes and shapes of the polygonal structures are dictated by the areal density of nucleation^35^. The higher the nucleation density, the smaller the textures.

**Figure 5:**
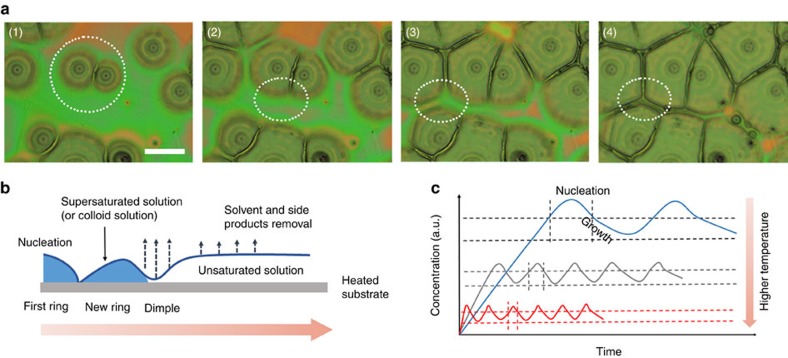
The morphology evolution and nucleation scheme of perovskites. (**a**) The selected *in situ* microcopy images of the perovskite films heating at 100 °C. The interval time is 0.02 s. The scale bar is 50 μm. The images of the whole nucleation and crystal growth were offered in Supplementary Fig. 12 in Supporting Information. (**b**) The scheme of the periodic and rhythmic crystallisation. (**c**) The schematic diagram of nucleation and growth with time based on various heating temperatures.

Figure 5a shows a series of *in situ* optical microscope images of the ripple formation (details are shown in Supplementary Fig. 12 with 0.02 s sampling time interval). Unlike polymeric spherulites that arise from the radial growth of crystalline lamellae emanating from a nucleation point, the banding in the perovskites are more closely related to the Lisegang rings^38^, resulting from a periodic precipitation reaction. The wavelength of the banding was found to decrease with increasing processing, as seen from the 120 °C thin films (Supplementary Figs 10 and 13). The periodic patterns observed in the perovskite crystallisation are a complex interplay between solution flow, solute diffusion, the solvent extraction and the crystal growth. When the perovskite solution is deposited on an elevated heating substrate, the evaporation process is more rapidly. Once the solution supersaturates, the nucleation occurs, and the crystal growth followed by. A rapid removal of solvent near the growth front induces liquid convection that carries solute to the crystal face to allow crystallisation to occur, depleting the volume in front of the growth front. This crystallisation-depletion mechanism^39^,^40^ gives rise to a concentration gradient and a periodic crystallisation of the perovskite, as shown schematically in Fig. 5b,c. AFM images in Fig. 4b show that the amplitudes of the bands decreased with the increasing temperature, which would be consistent with a more rapid diffusion of materials to the growth front and a slower crystallisation growth rate^41^,^42^. By increasing the precursor solution volume to decrease the crystal growth, the periodicity of the bands decreased, whereas the amplitudes increased (Supplementary Fig. 14). To increase the crystal growth by rapidly removing the solvent within seconds using HAQ, the morphology was dominated by small crystalline grains, hundreds of nanometres in size, similar to the plateau region in the centre of the banded texture. A root mean square roughness of HAQ film was 4.7 nm, whereas the roughness of the printing films are 110.5 nm (60 °C), 119.3 nm (80 °C), 59.2 nm (100 °C), 38.2 nm (120 °C). The low roughness of HAQ perovskite film indicated the smoothness quality and could be readily used in device fabrication. In addition, we also fabricated the perovskite films via traditional spin coating or simple drop casting method to compare with the slot die printing perovskites. Detailed experiments and discussion are provided in supporting Supplementary Fig. 15 and Supplementary Note 2.

We fabricated the inverted planar heterojunction perovskite solar cells^43^ (PSCs) based on this research (Supplementary Fig. 16) to investigate the film quality, and remarkable device performances were achieved (Supplementary Fig. 16-20 and Supplementary Note 3). The champion PCE of 15.1% for the small-area (0.09 cm^2^) PSC (Supplementary Fig. 18) and champion PCE of 11.6% for the large-area (1.00 cm^2^) PSC (Supplementary Fig. 19) were achieved. These results imply that the perovskite films studied in this research have comparable optoelectronic properties as the films fabricated with traditional spin-coating process^43^, indicative of great potential for large-area devices based on printing fabrictaion. Consequently, the banded texture of the perovskite films is dictated by nucleation, which decreases with increasing temperature, hence the increase in the size of the structures, and a radial growth rate of the crystals, G, with dimension of length/time, and the diffusion of materials to the growth front, D, with dimension (length)^2^/time. The ratio of D/G defines a length that characterizes the period of the banded structures. This feature is similar to rhythmic crystallisation seen in soft materials^41^,^42^.

## Discussion

In summary, this study revealed the material transformation and morphology formation of perovskites from precursor solution to the polycrystalline film over relevant temperature and time scales. We utilized some advanced *in situ* characterisation techniques to address an important challenge in perovskite material research. We identified a perovskite crystal intermediate, comprised of an octahedral [PbI_6_]^4−^ centre surrounded by cooperative ions. The close packing of these ion cage species led to the formation of perovskite crystallites, which then deposited onto the supported substrate to form photoactive thin film. The nano-assemble model via intermediate [PbI_6_]^4−^ centre nanoparticles was proposed to understand perovskites formation at a molecular level. The growth kinetics in mesoscopic structure are dictated by the rate of solvent evaporation and chemical constituent diffusion, which also gives a handle of morphology control. Elevated-temperature annealing led to a periodic crystallisation growth habit, forming concentric ring patterns upon the crystallisation-depleting mechanism. And increasing the rate of solvent evaporation through the HAQ process, the morphology was kinetically trapped, resulting in a smooth film state that was better suited for thin-film optoelectronic devices. Importantly, the current results show the interesting perovskite material science at nano-to-meso scales, offering new perspectives to tune the crystal kinetics and material transformation, and pave an avenue to make high-quality perovskite thin films for wide optoelectronic device applications.

## Methods

### Materials

MAI (CH_3_NH_3_I) was synthesized from CH_3_NH_2_ and HI based on the reported literature^44^. Lead acetate (Pb(Ac)_2_) was purchased from Sigma Aldrich and was dehydrated before use^45^. Poly(3,4-ethylenedioxythiophene):poly(styrenesulfonate) (PEDOT:PSS, PVP AI4083) was purchased from Heraeus Clevios. [6,6]-phenyl C_61_-butyric acid methyl ester (PC_61_BM) was purchased from C-Nano Tech. 2,9-dimethyl-4,7-diphenyl-1,10-phenanthroline was purchased from Alfa Aesar. All the liquid reagents, including DMF, chlorobenzene (CB) and chloroform (CF) were purchased from Acros and used as received.

### Device fabrication

The devices were manufactured on the pre-patterned glass/ITO substrates (3.0 inch × 1.0 inch, 20 Ω □^−1^), then ultrasonically cleaned with the following sequential steps: diluted detergent, deionized water, acetone and isopropanol. Before spin-coating, the glass/ITO substrates were treated by UV-Ozone for 15 min. Then PEDOT:PSS was spin-coated onto the ITO substrates at 4000, rpm for 30 s and subsequently annealed at 135 °C for 20 min in ambient atmosphere. 0.4 M lead acetate (Pb(Ac)_2_) and 1.2 M MAI were dissolved in anhydrous DMF to prepare for the perovskite precursor solution. For the spin-coating samples, the precursor solution was spin-coated at 4000, rpm for 60 s, and then the substrates were annealed at 80 °C for 5 min. For the perovskite through slot die printing method, coating was carried out at the optimized speed of 30 mm s^−1^ with the optimized solution pump speed of 70 μl min^−1^, the distance between the slot die head to the substrate was set at from 0.2 mm to 0.4 mm. Then the substrate with the wet perovskite precursor film was transferred under the hot nitrogen (∼180 °C) operated by a Deluxe heat gun and removed quickly as soon as the yellow wet film became dark brown dry film. For small-area devices, the perovskite films cooled down to room temperature, the PC_61_BM (20 mg ml^−1^ in CB and CF at the volume of 4:1) solution was printed on the top of perovskite layer. For the PC_61_BM, the coating speed is 10 mm s^−1^ with the solution pump speed of 70 μl min^−1^, the distance between the slot die head and the substrate was set at 0.3 mm. To achieve uniform PC_61_BM layer in large area (1.00 cm^2^) and make the PC_61_BM layer fully cover the perovskite layer, the PC_61_BM (20 mg ml^−1^ in CB) solution was spin-coated on the top of perovskite layer at 1000, r.p.m. for 30 s. The whole sampling process was performed in a N_2_-filled glove box. Afterwards, 10 nm-thick 2,9-dimethyl-4,7-diphenyl-1,10-phenanthroline and 100 nm-thick metal silver electrode were thermally evaporated in the vacuum chamber with the base pressure of <4 × 10^−4^ Par through a shadow mask.

### Characterisation of PSCs

The cells (active area: 0.09 cm^2^∼1.00 cm^2^) were irradiated under 100 mW cm^−2^ by a 150 W class AAA solar simulator (XES-40S1, SAN-EI) with an AM 1.5G filter. The light intensity was calibrated by a KG-5 silicon diode. The IPCE spectra were measured using a lock-in amplifier coupled with a monochromator (Crowntech, Qtest Station 2000, USA). The light intensity of the monochromator was calibrated by a standard monocrystalline Si photovoltaic cell. The champion devices were tested using an aperture mask (small area of 0.09 cm^2^) to make an accurate illumination area.

### Wide angle grazing incidence X-ray scattering measurements

GIXD measurements were conducted on beamline 7.3.3 at Advanced Light Source, Lawrence Berkeley National Laboratory. The wavelength of X-ray was 1.240 Å, and the scattering intensity was detected by a PILATUS 2M detector. The mini slot die instrument was installed in helium box, and a surveillance camera was used to monitor the slot die head for the solution flow. The substrate under the slot die head was pre-aligned and the incident angle was adjusted to 0.5° for the *in situ* probe. The slot die printer was fixed at an accurate distance from the substrate to ensure the uniform quality of the film and the reproducibility. The exposure time was 5 s in single mode for each frame when the substrate was in room temperature, whereas the typical exposure time was 0.1 s in burst mode when the substrate was heated at 60∼120 °C. All the Si substrates were covered with PEDOT:PSS. The 2D GIXD images were sector averaged using Nika software package. The integrated peak area, the FWHM of the peaks and the crystal size were performed using IGOR software.

### Other characterisations

The morphological SEM images were obtained through FESEM, Zeiss, ULTRA-55. The AFM images were collected in tapping mode by the Dimension 3100 AFM combined with NanoScope 3D systerm, Veeco Instruments Inc. The FTIR spectra were taken in transmission mode on a Thermal Scientific Nicolet 6700 spectrometer, which was equipped with a deuterated triglycine sulfate detector. Samples for FTIR measurements were prepared onto KBr substrates (International Crystal Laboratories). The optical microcopy images were collected by Zeiss Axioskop 2, F5 plus compound microscope integrated with a thermocouple module.

### Data availability

All relevant data are available from the corresponding authors upon reasonable request.

## Additional information

**How to cite this article:** Hu, Q. *et al. In situ* dynamic observations of perovskite crystallisation and microstructure evolution intermediated from [PbI_6_]^4−^ cage nanoparticles. *Nat. Commun.* **8,** 15688 doi: 10.1038/ncomms15688 (2017).

**Publisher’s note:** Springer Nature remains neutral with regard to jurisdictional claims in published maps and institutional affiliations.

## Supplementary Material

Supplementary Information Supplementary Figures, Supplementary Notes and Supplementary References
