# Supplementary material for: In situ dynamic observations of perovskite crystallisation and microstructure evolution intermediated from [PbI6]4− cage nanoparticles
Source: Nat Commun. 2017 Jun 21;8:15688. doi: 10.1038/ncomms15688 (PMC5482054; doi:10.1038/ncomms15688)
Supplement: Supplementary Information — Supplementary Figures, Supplementary Notes and Supplementary References [file ncomms15688-s1.pdf]

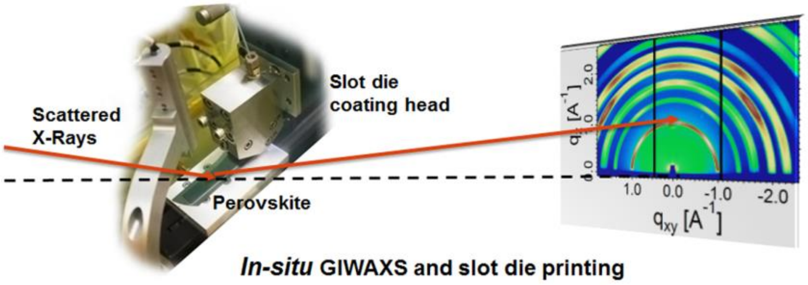

Supplementary Figure 1 | Scheme image of GIXD set-up. The scheme image of slot die printing system combined with grazing incidence X-ray diffraction (GIXD) set-up.

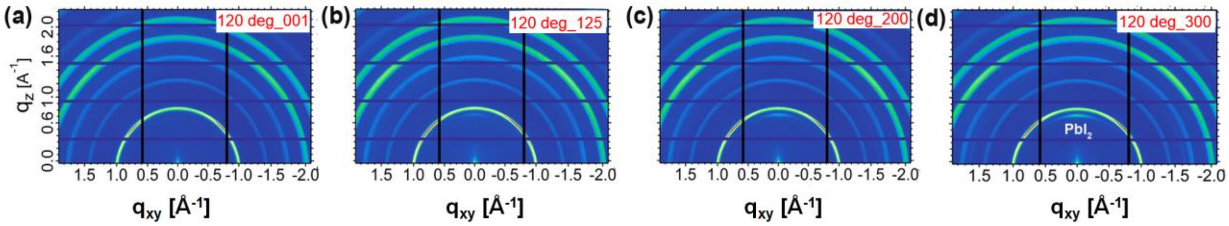

Supplementary Figure 2 | 2D GIXD images of perovskite films. The selected 2D GIXD images of perovskite films along with time (frames) upon annealing at 120 ℃ (deg).

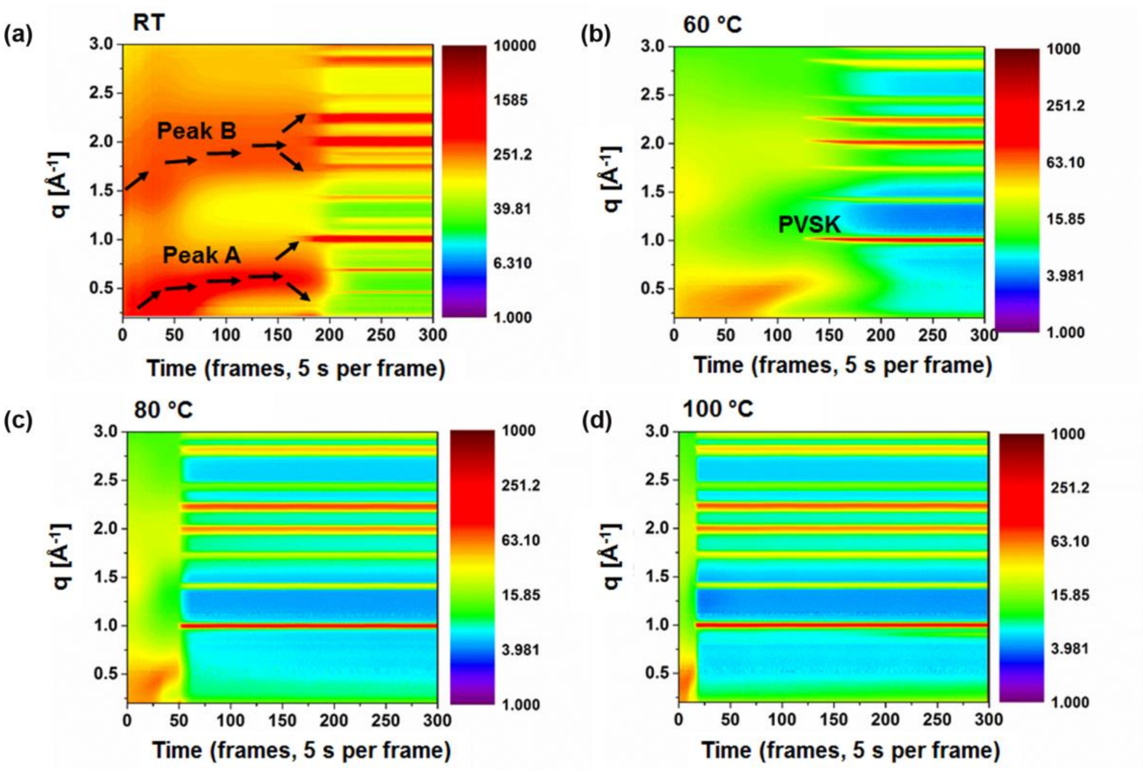

Supplementary Figure 3 | *In-situ* integral GIXD profiles of printing perovskites. Image plots of the integrated scattered intensity (color scale) versus q and time (frames) at different heating temperatures: (**a**) room temperature (RT), (**b**) 60 ℃, (**c**) 80 ℃ and (d) 100 ℃. The arrows in figure exhibit the evolution of the peak position from the initial precursor solution to the final perovskite products.

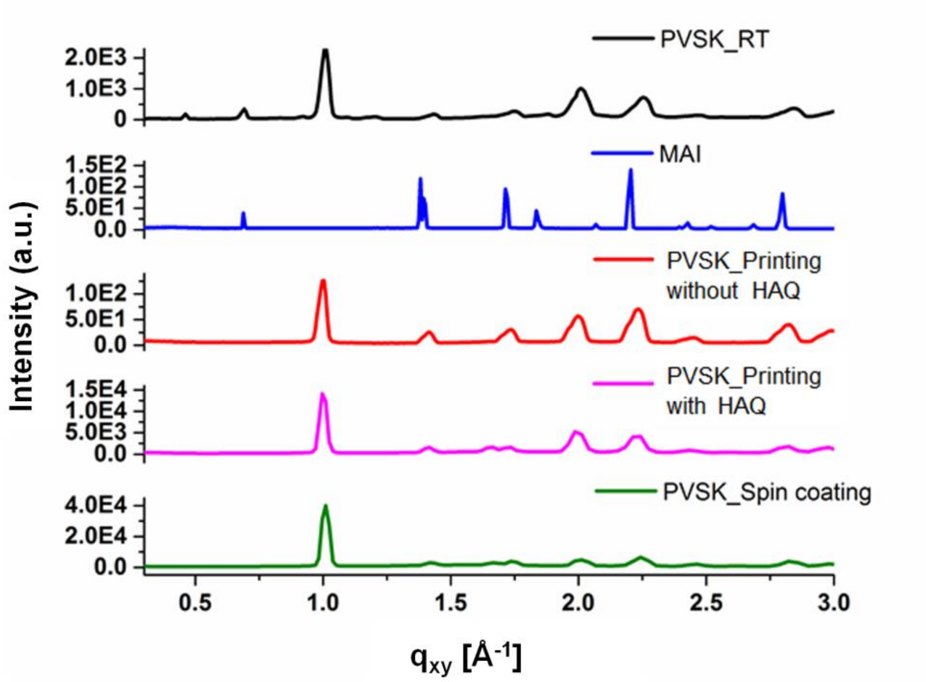

Supplementary Figure 4 | The GIXD data of different samples in this study. The samples are: perovskite ($CH_3NH_3PbI_3$) film at room temperature (black line), MAI (methylammonium iodide) powder (blue line), the perovskite film obtained from the slot die printing with substrate heated at 80 ℃ (red line), the slot die printing perovskite film treated with hot air quenching (HAQ) process (magenta line) and the perovskite film fabricated with spin coating method (olive green line).

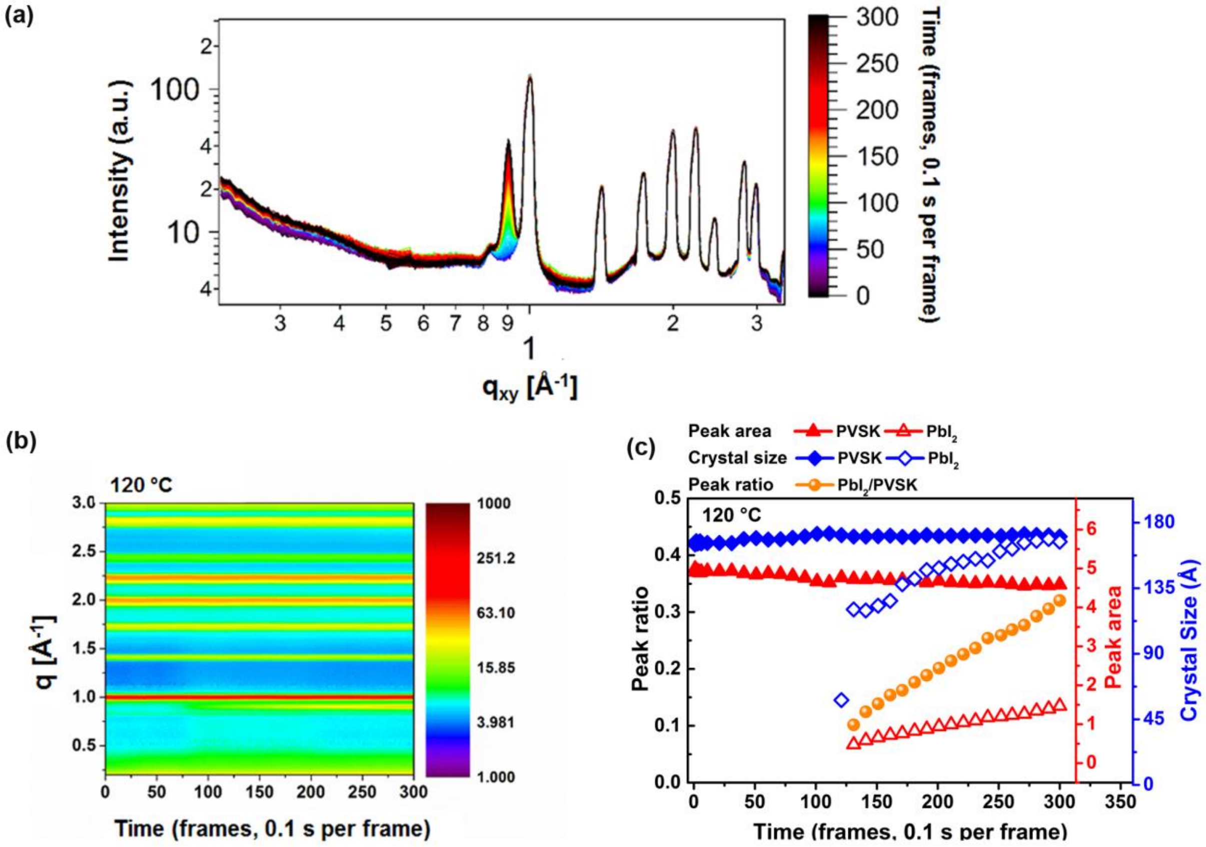

Supplementary Figure 5 | The crystallization and degrading process of perovskite film annealing at 120 ℃. (**a**) *In-situ* 1D sector X-ray profiles integrated from 2D GIXD shown in Supplementary Fig. 2, (**b**) Image plots of the integrated scattered intensity (color scale) versus q and time, (**c**) The diffracting peak intensity, the peak area and the crystal size of the characteristic peaks. The final peak area ratio between PbI_2 and perovskite was increased from 5.34% at 100 ℃ to 32.04% at 120 ℃.

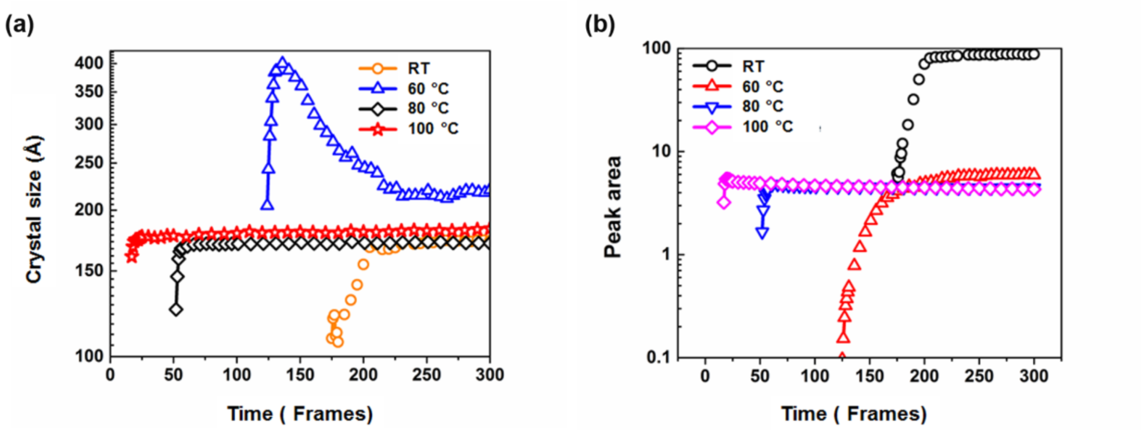

Supplementary Figure 6 | The analysis of crystallization process of the printing perovskites.

The crystal size (**a**) and peak area (**b**) of the perovskite (100) plane peak at different temperatures.

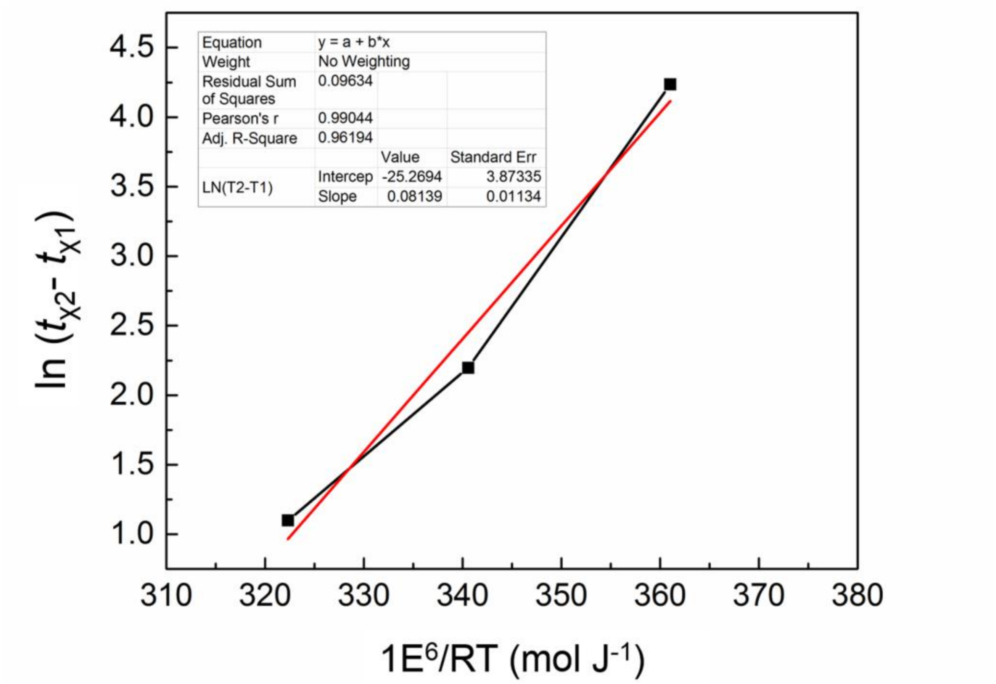

Supplementary Figure 7 | Plot constructed from Johnson-Mehl-Avrami model. For the equation "ln ($t_{\chi2}$ - $t_{\chi1}$) = E_a/RT-ln κ_0 + ln ($\beta_{\chi2}$ - $\beta_{\chi1}$)", to extract the effective activation energy (E_a), the slope of the data plotted as ln ($t_{\chi2}$ - $t_{\chi1}$) versus 1/RT is E_a. The slope of the line was ~81.39 kJ mol^{-1}. Here, the temperatures were 60 ℃, 80 ℃, 100 ℃, respectively, and χ2 was ~1.00, χ1 was ~0.58.

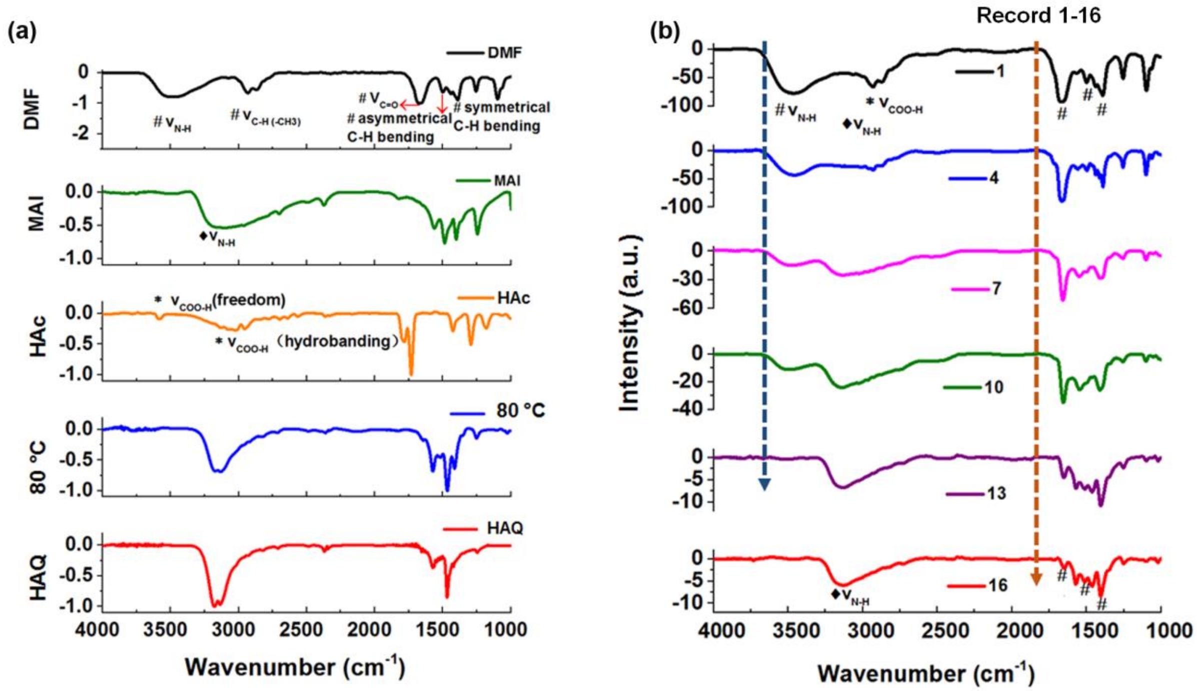

Supplementary Figure 8 | The Fourier transform infrared (FTIR) spectroscopy. (**a**) The FTIR spectra of different chemical materials. (**b**) The *in-situ* FTIR spectra of the perovskite precursor at room temperature along with time (as the arrows show), and the record interval time is ~60 s.

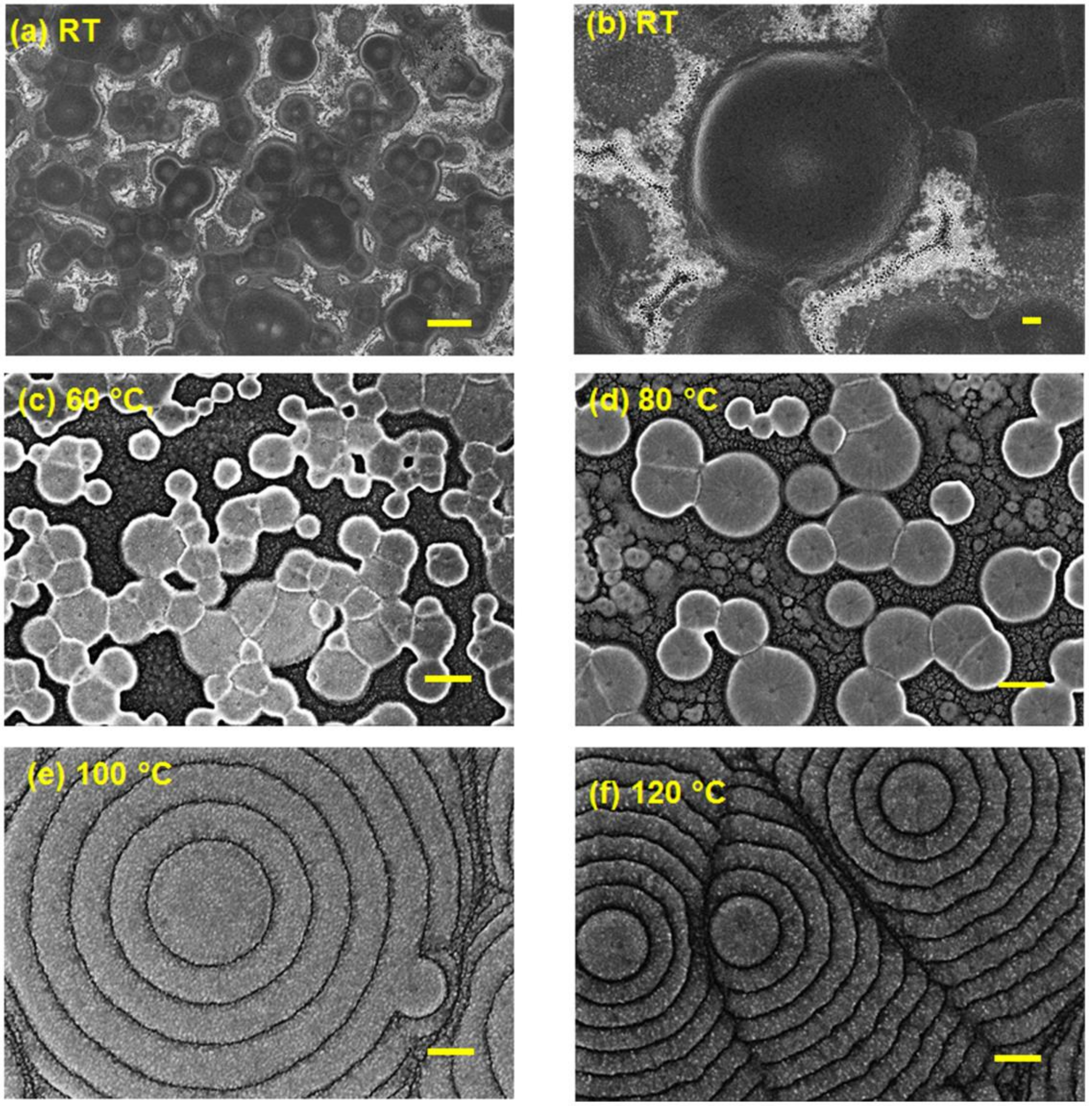

Supplementary Figure 9 | The scanning electron microscope (SEM) images of printing perovskites films. Perovskite films printed on various heating temperatures: (**a**, **b**) RT, (**c**) 60 ℃, (**d**) 80 ℃, (**e**) 100 ℃, (**f**) 120 ℃, the slot die coating speed was 30 mm s^{-1}, the distance between the slot die head and the substrate was 0.2 mm. The pump speed was 70 μl min^{-1} for all the printing films in this paper without special illustration. The scale bar in Fig. a is 20 μm while the scale bars in Fig. b-d are 2 μm.

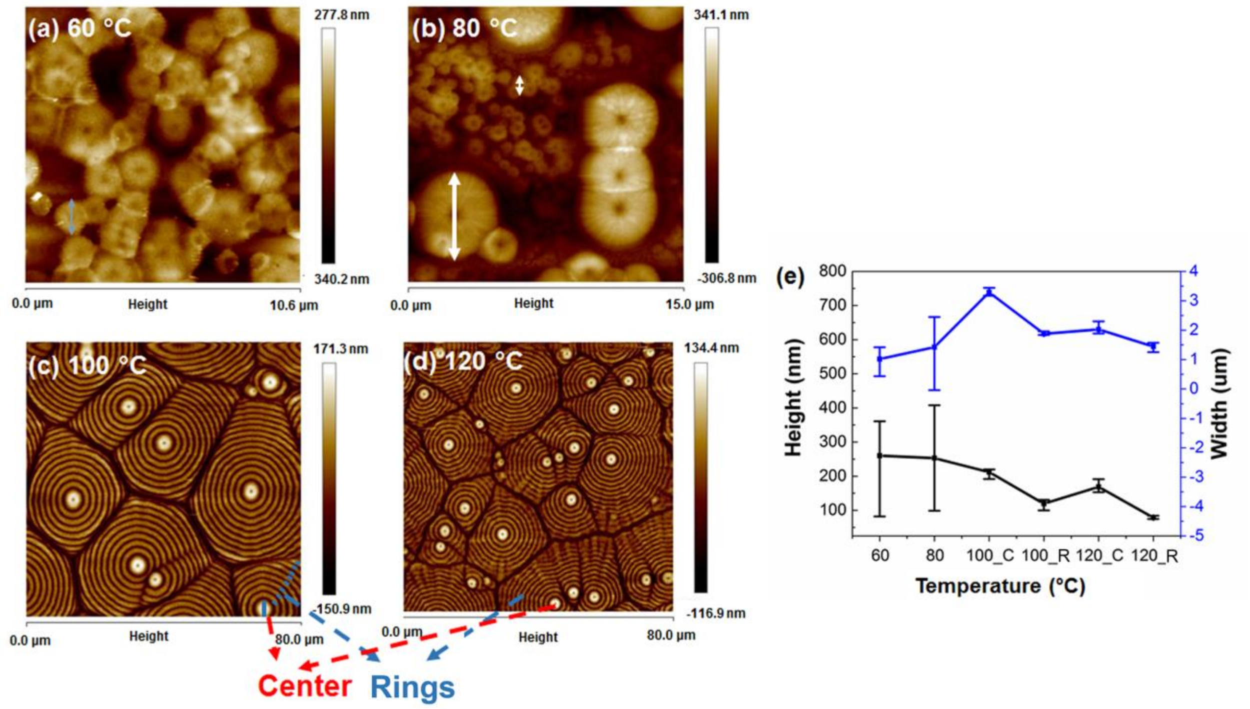

Supplementary Figure 10 | The topographic atomic force microscope (AFM) images of printing perovskites films. The AFM images of printing perovskite films based on various heating temperatures: (**a**) 60 ℃, (**b**) 80 ℃, (**c**) 100 ℃, (**d**) 120 ℃, while slot die coating speed was 30 mm s^{-1}, the distance between the slot die head and the substrate was 0.2 mm. (**e**) The height and width statistics of the rings at different temperatures. The 100_C and 120_C are abbreviated for the center rings of the concentric domains at 100 ℃ and 120 ℃. The 100_R and 120_R are abbreviated for the side rings of the concentric domains at 100 ℃ and 120 ℃. The measured root mean square roughness was only 4.7 nm while the roughness of the printing films are 110.5 nm (60 ℃), 119.3 nm (80 ℃), 59.2 nm (100 ℃), 38.2 nm (120 ℃).

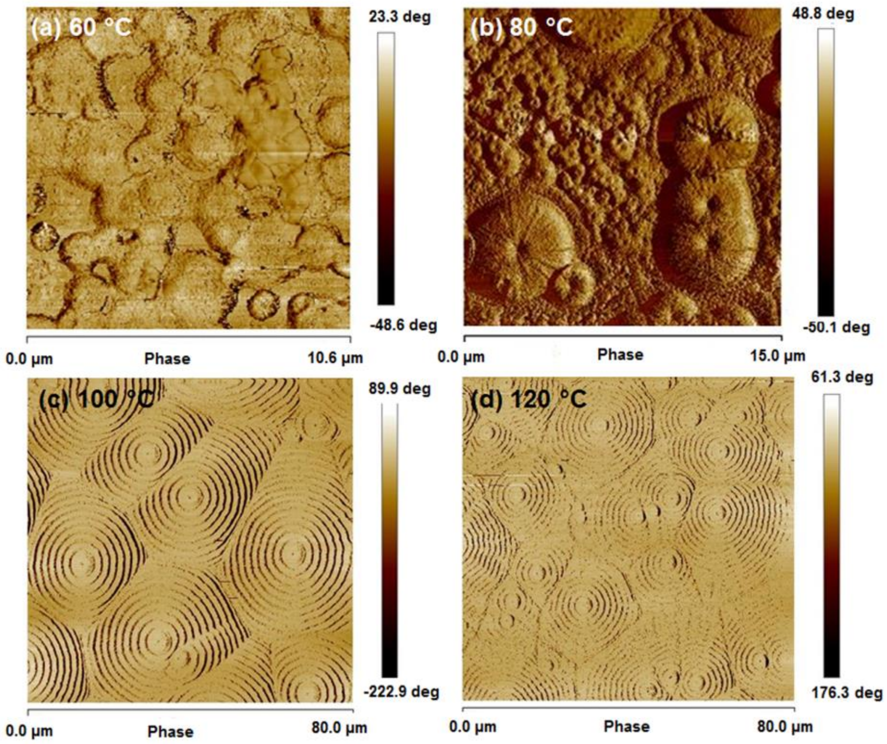

Supplementary Figure 11 | The phase AFM images of printing perovskites. The phase AFM images of the printing perovskite films based on various heating temperatures: (**a**) 60 ℃, (**b**) 80 ℃, (**c**) 100 ℃, (**d**) 120 ℃, while the coating speed was 30 mm s^{-1}, the distance between the slot die head and the substrate was 0.2 mm.

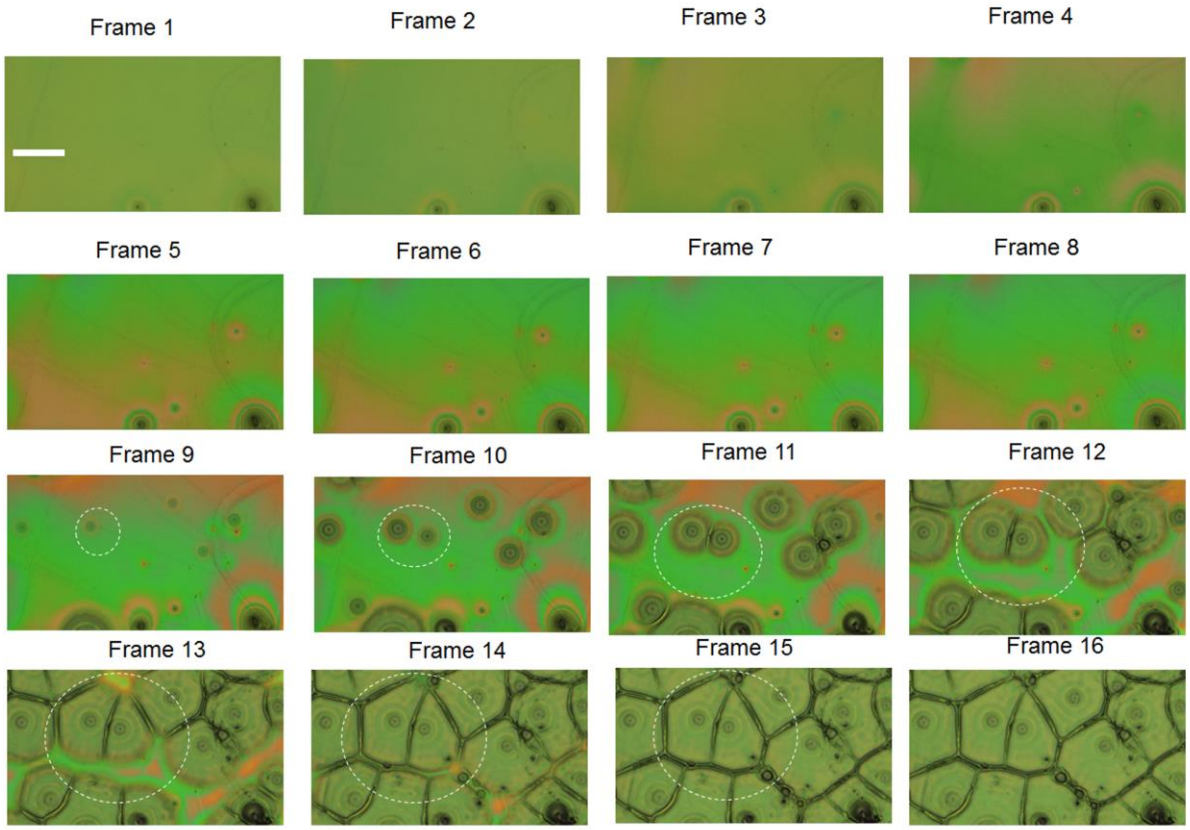

Supplementary Figure 12 | *In-situ* microcopy images of the perovskite film. The optical images monitored the perovskite film morphology evolution from precursor solution to dried film *in situ* while heating at 100 ℃. The interval time between frames is 0.02 s. The scale bar is 50 μm.

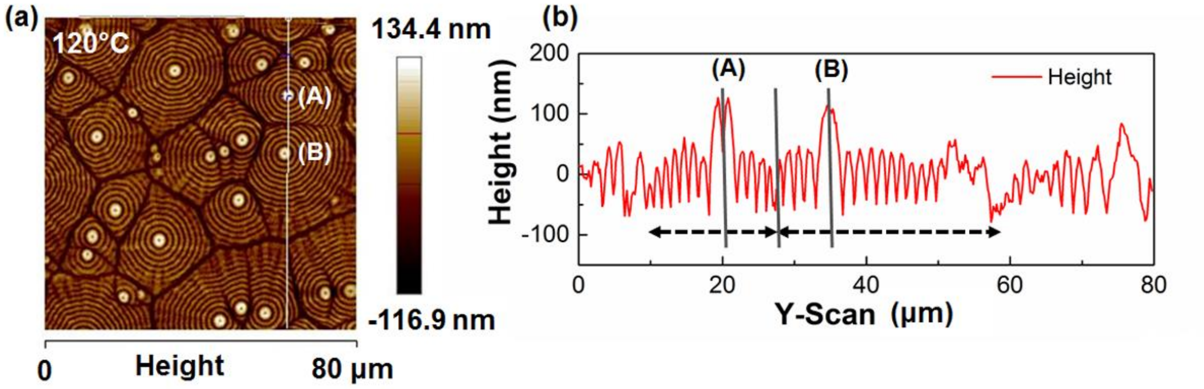

Supplementary Figure 13 | Topographical AFM image with cross-sectional line profile. (**a**) The topographical AFM image of the perovskite film under the condition of 120 ℃, 30 mm s^{-1}, 0.2 mm. (**b**) The cross-sectional line profile of the perovskite rings based on the height image in Fig. a.

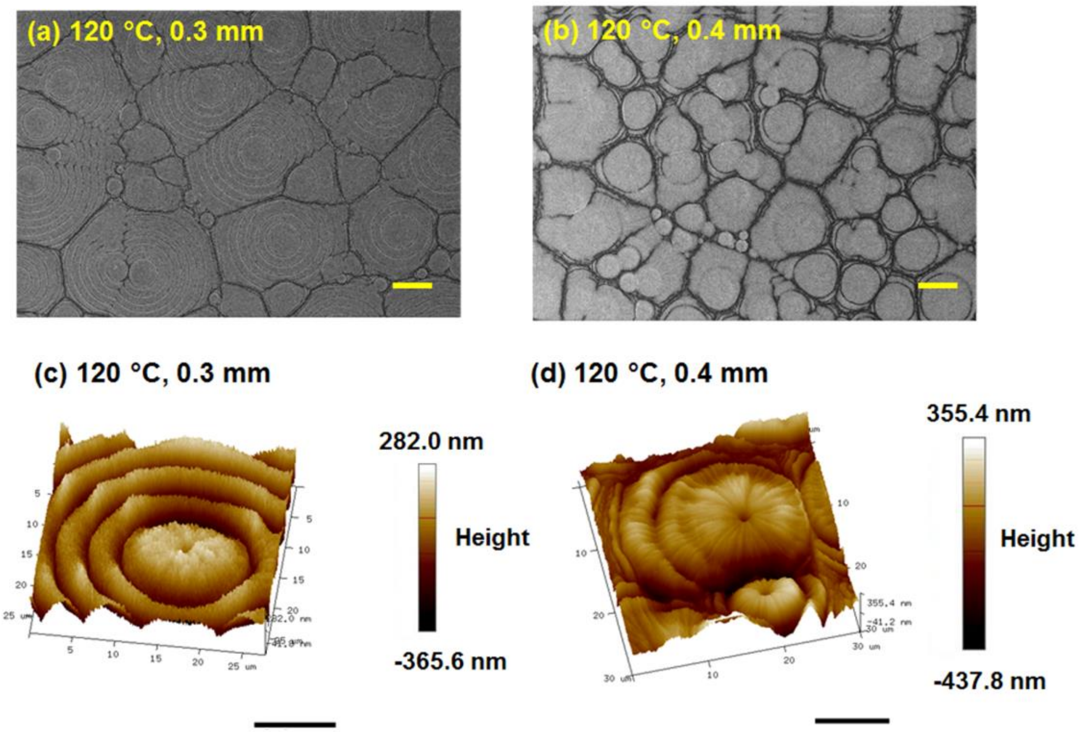

Supplementary Figure 14 | The morphologies of perovskite films printed under different conditions. The SEM images of perovskite films with the conditions of (**a**) 120 ℃, 30 mm s^{-1}, 0.3 mm, (**b**) 120 ℃, 30 mm s^{-1}, 0.4 mm and the corresponding AFM images: (**c**) 120 ℃, 30 mm s^{-1}, 0.3 mm, (**d**) 120 ℃, 30 mm s^{-1}, 0.4 mm. The scale bars in Fig. a and Fig. b are 20 μm while the scale bars in Fig. c and Fig. d are 6.0 μm.

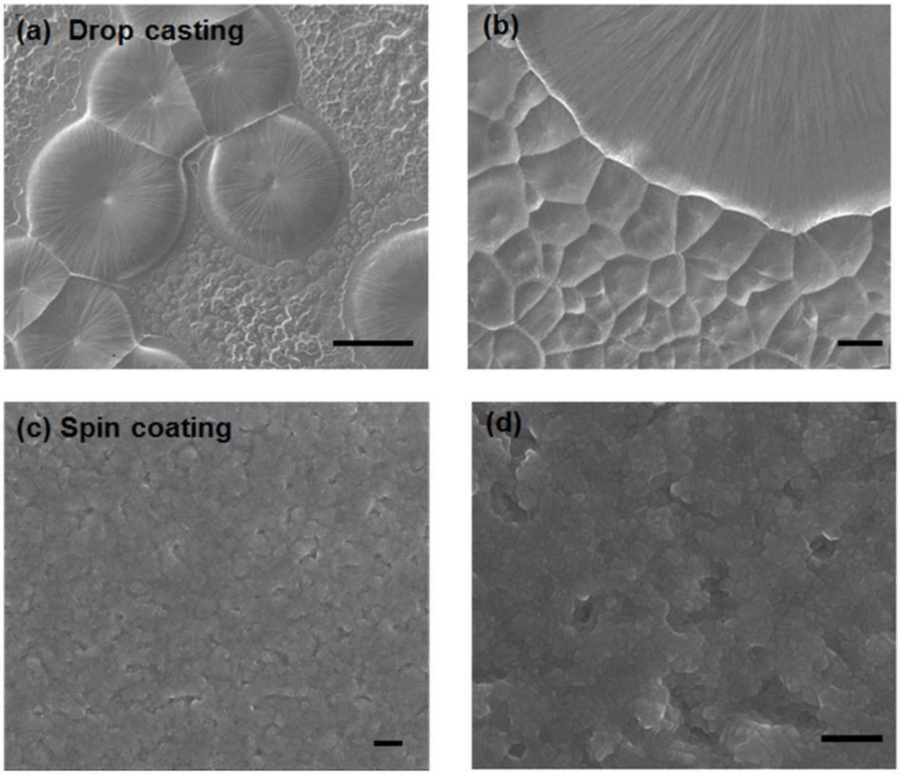

Supplementary Figure 15 | The morphologies of the perovskite films proceeded from different procedures. The SEM images of the perovskite films proceeded from (**a**, **b**) drop coating and (**c**, **d**) spin coating. The scale bars of Fig. a-d are 50 μm, 10 μm, 1 μm and 500 nm, respectively.

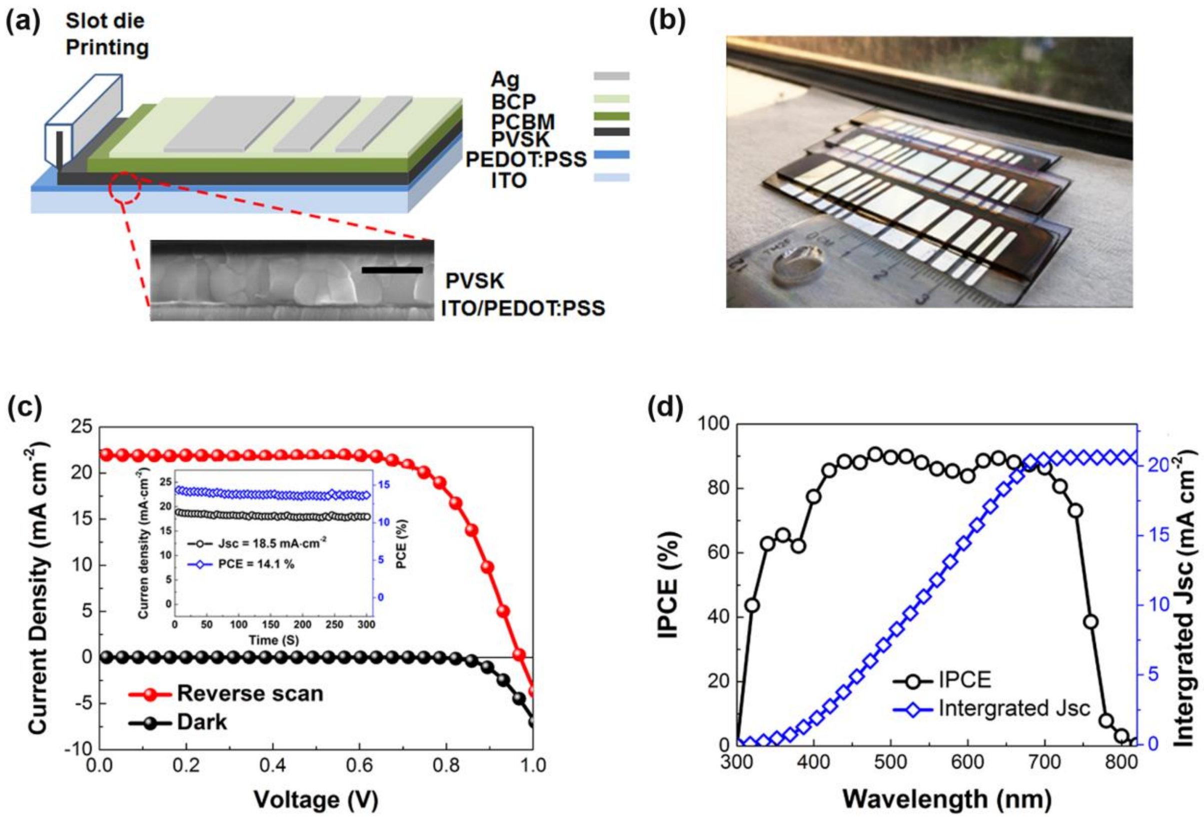

Supplementary Figure 16 | Photovoltaic characterization of printing perovskite solar cells (PSCs). (**a**) The device structure of the PSCs based on slot die printing process, insert: the cross-sectional SEM image of the perovskite film on the ITO/PEDOT:PSS substrate (scale bar, 500 nm). (**b**) The picture of the devices in this study. (**c**) The *J-V* curve of the champion device (*PCE* = 15.1%) under AM 1.5G illumination of 100 mW cm^{-2}, insert: the steady-state photocurrent and output efficiency at the maximum power point. (**d**) The IPCE spectrum of the champion device and the integrated current density based on the IPCE.

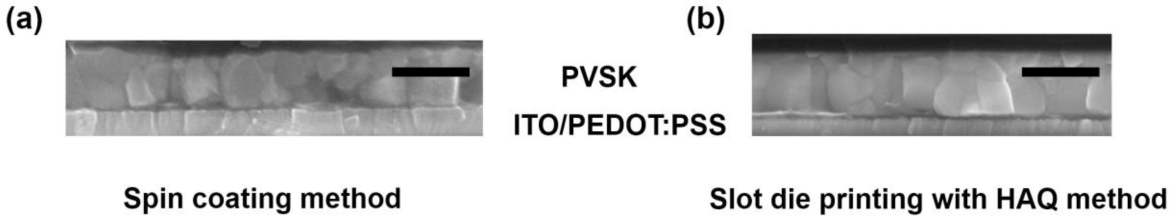

Supplementary Figure 17 | The cross-sectional SEM images of the perovskite films. The cross-sectional SEM images of the perovskite film through different methods on ITO/PEDOT:PSS substrates: (**a**) spin coating method (**b**) slot die printing with HAQ method. The scale bars in are both 500 nm.

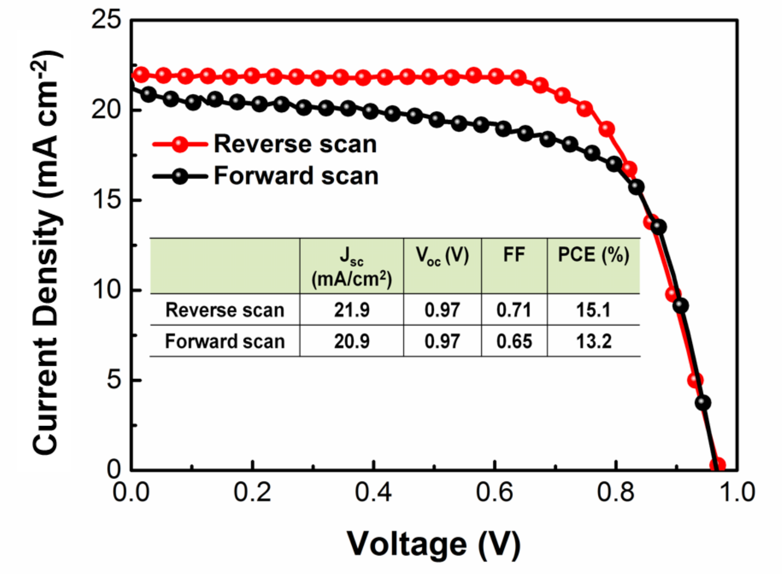

	J_{sc} (mA/cm²)	V_{oc} (V)	FF	PCE (%)
Reverse scan	21.9	0.97	0.71	15.1
Forward scan	20.9	0.97	0.65	13.2

Supplementary Figure 18 | The *J*-*V* curves of the champion PSC on small area. The *J*-*V* curves of the champion device of small area (0.09 cm^2) based on different scan directions under AM 1.5G illumination of 100 mW cm^{-2}.

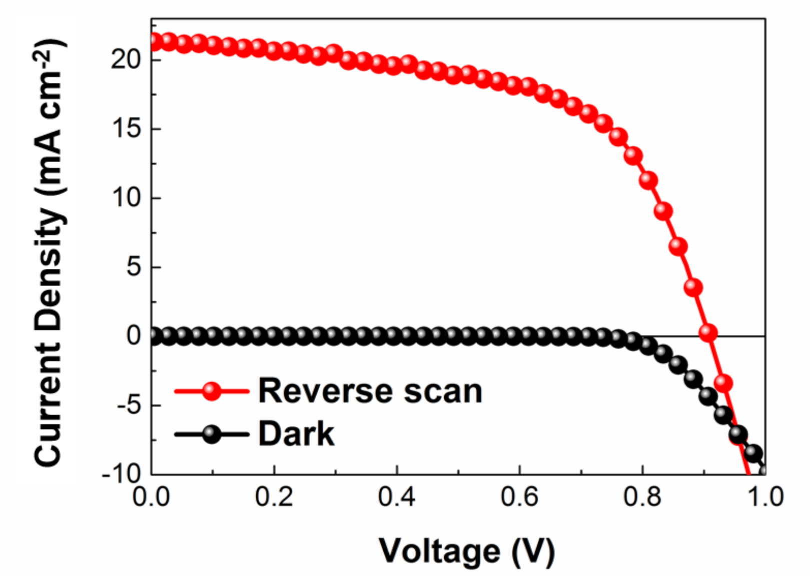

Supplementary Figure 19 | The *J-V* curves of the champion PSC on large area. The *J-V* curves of the champion device of larger area (1.00 cm^2) measured under AM 1.5G illumination of 100 mW cm^{-2}. A *PCE* of 11.6% under standard illumination with J_{sc}, V_{oc}, and *FF* reaching values of 21.4 mA cm^{-2}, 0.92 V and 0.59 achieved.

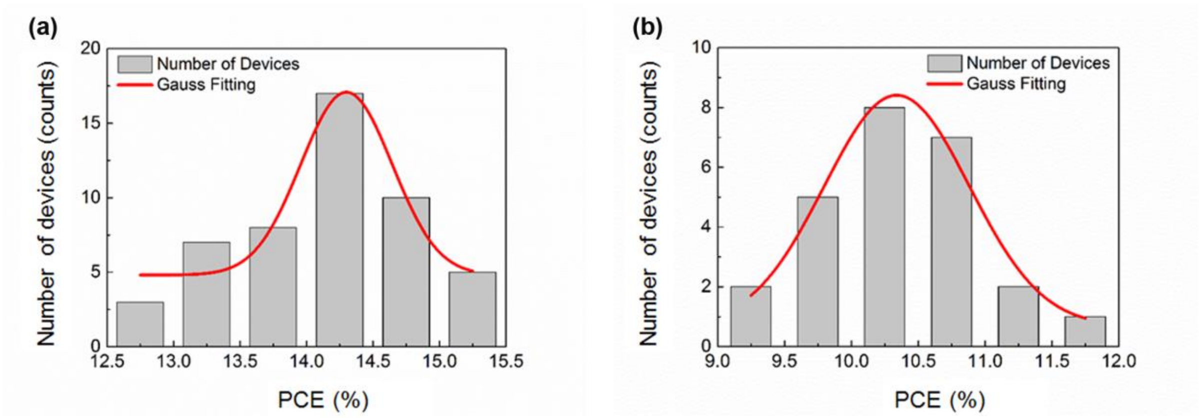

Supplementary Figure 20 | Histograms of the device efficiencies. Histograms of the device efficiencies (*PCE*s) in small area (0.09 cm^2) and larger area (1.00 cm^2) measured from 50 and 25 devices, separately. The Guassian distribution fitting curves were also provided.

Supplementary Note 1 | Perovskites crystallization and structure transformation.

In Supplementary Fig. 6, Gaussian-function fitting was applied to determine the peak position, peak area and the full width at half maximum (FWHM) of the special peaks through Igor software. The crystal size was obtained by Debye-Scherer equation, $D = \kappa\lambda/\beta\cos(\theta)$, where D is the mean size of the ordered crystalline domains (which could be equal to or smaller than the grain size), κ is a dimensionless shape factor, λ is X-ray wavelength, β is the line broadening at half of the maximum intensity (FWHM), θ is Bragg angle. At RT, The final crystal size was ~200 Å. The crystal size increased along with the growth of the crystal. However, at 60 ℃, the crystal size firstly increased to 399.0 Å from frame 124 (12.4 s) to frame 137 (13.7 s) and then deceased to 215 Å at frame 226.0 (22.6 s). The crystal size reduction arises, more than likely, from the introduction of defects, such as the DMF, incorporated within the crystal and then removed under elevated annealing. At 80 ℃, perovskite crystals formed very rapidly and then stabilized at 171.0 Å. The crystal was quite stable and, thus, DMF was not incorporated within the crystal at 100 ℃. The perovskite crystal formation was completed in several seconds and the final crystal size was about 180.0 Å. The peak area was summarized from the Gaussian-function fitting of the (100) peak. The peak area at different temperatures have similar increasing trend, indicating the similar growth process of the perovskites. The perovskite was the only final product for the printing film heated at from 60 ℃ to 100 ℃. The peak areas were applied in the Johnson-Mehl-Avrami model to calculate the effective activation energy.

Supplementary Note 2 | Perovskite films processed from different procedures.

Supplementary Fig. 15 shows the morphologies of the perovskite films proceeded from different procedures including drop coating and spin coating. The results for different stages of

the perovskite crystallization (Fig. 3 in the maintext) in slot die printing from precursor solution to colloidal intermediated composition, to the dried polycrystalline perovskite film and to degrade to the lead iodide are applicable to the simple drop-casting or common spin-coating procedure. The fundamental chemistry will not change with different process. From the previous XRD results reported by Dr. Zhang Wei and his colleagues[1], we can conclude that the wet film after spin coating has similar diffraction peak positions with the slot-die-printing film at room temperature, indicating the formation of intermediated $MAI \cdot PbI_2 \cdot DMF$ composition, which is the similar process of stage 1 in our study. Supplementary Fig. 4 and 8 show that the final dried films of spin coating and slot die printing annealed at 80 ℃ have almost the same XRD and FTIR spectra, proving the same final product, namely, the same stage 2. When the perovskite film are annealing at high temperature and prolonged time, the $CH_3NH_3PbI_3$ could decompose to PbI_2, which is the stage 3. In summary, the formation mechanism and fundamental chemistry is universal to different procedure.

As Supplementary Fig. 15 shows, the morphology could be various with different procedures. The drop casting leads to large domain size of tens micrometer, which is similar to slot die printing films at low temperature (Fig. 4 in the maintext), while the domain size is much larger than the grain size of hundreds nanometer on the spin-coating film. This major differences in morphology are resulted from the different drying kinetics. A slow solution drying in drop casting leads to prolonged crystal growth and thus form large spherulite crystals. Increasing drying kinetics using slot die processing under elevated temperatures could lead to a balanced crystallization between material diffusion and solvent evaporation, then obtaining a rhythmic crystallization process. In spin coating, the dramatically faster solvent removal could kinetically trap the crystallization in an earlier stage and thus reduces crystal sizes.

Supplementary Note 3 | Perovskite solar cells based on slot die printing.

The optoelectronic properties of the perovskite films strongly depend on the crystallinity and morphology. We fabricated the planar heterojunction perovskite solar cells (PSCs) based on this research to investigate the film quality and characterize the device performance. PSCs were fabricated using a device structure of ITO/PEDOT:PSS/$CH_3NH_3PbI_3$/PCBM/BCP/Ag[2] (Supplementary Fig. 16). The PEODT:PSS and $PC_{61}BM$ were selected as the hole transport and electron transport layers, respectively. The $CH_3NH_3PbI_3$ perovskite photoactive layer was processed by HAQ with a ~350 nm thickness. The inset of Supplementary Fig. 16a shows that large grains, hundreds of nanometers in size were formed in lateral direction, which are larger than those observed in conventional spin-coating fabrication (Supplementary Fig. 17). The grain size in the vertical direction to the perovskite film is comparable to film thickness, thus efficient carrier transport can be achieved. Supplementary Fig. 16b shows a picture of the perovskite solar cell fabricated in this study. The champion *PCE* of 15.1% for the small area (0.09 cm^2) PSC was achieved with a short circuit current density (J_{sc}) of 21.9 mA cm^{-2}, an open-circuit voltage (V_{oc}) of 0.97 V and a fill factor (*FF*) of 0.71 under AM 1.5G illumination of 100 mW cm^{-2}. A stabilized *PCE* of 14.1% was obtained at the maximum power point (V = 0.76 V, J_{sc} = 18.5 mA cm^{-2}) as shown in Supplementary Fig. 16c. The incident photon to current conversion efficiency shown in Supplementary Fig. 16d was examined and the integrated current density was 20.6 mA cm^{-2}, which was in good agreement with the short-circuit current density. Large-area devices with active area 1.00 cm^2 were also fabricated basing on the slot die printing and HAQ method. A champion *PCE* of 11.6% under standard illumination with J_{sc}, V_{oc}, and *FF* reaching values of 21.4 mA cm^{-2}, 0.92 V and 0.59 were achieved (Supplementary Fig. 19). In addition, we also fabricated the device based on the slot die printing without HAQ method, however, the device

performances were very poor. That is because of the non-continuous and rugged film morphology. As Fig. 4 in the maintext and Supplementary Fig. 10 show, the height variation of these periodic structure at the printing perovskite film surface, i.e. the amplitude, could be as large as hundreds of nanometers to micrometer. On one hand, there must be uniform $PC_{61}BM$ layer as thick as micrometer to cover the whole perovskite film, on the other hand, it is not easy to achieve high device performance based on such rough perovskite active layer even though the perovskite layer could be fully covered by $PC_{61}BM$.

In fact, from slot die printing to HAQ experiment, we are running experiment under the same controlling parameter and the same drying kinetics. We see by detailed morphology characterization and in situ crystal growth study that increase the drying speed of perovskite solution could suppress the rhythmic crystallization and form smooth films, which yield ideal morphology to fabricate solar cells. In morphology investigation and *in-situ* study, we have clearly elaborated their differences. We conclude that at ~80 ^{o}C printing temperature, film got dried within seconds, leaving us no much room to observe the morphology evolution details. HAQ method makes film dried even quicker, and we cannot conduct the *in-situ* experiment. Thus, we only provide static HAQ thin film morphology results. These results imply that the perovskite films via HAQ method in this research have comparable optoelectronic properties as the films obtained using traditional spin-coating fabrication. Furthermore, the uniform perovskite films could also have wider applications in other large-area optoelectronic devices based on slot die printing process and HAQ method.

Supplementary References

1. Zhang, W. *et al.* Ultrasmooth organic-inorganic perovskite thin-film formation and crystallization for efficient planar heterojunction solar cells. *Nat. Commun.* **6,** 6142 (2015).
2. Zhao, L. *et al.* High-performance inverted planar heterojunction perovskite solar cells based on lead acetate precursor with efficiency exceeding 18%. *Adv. Funct. Mater.* **26,** 3508-3514 (2016).
